# Global strategy to eliminate cervical cancer as a public health problem: are we on track?

**DOI:** 10.1016/j.eclinm.2023.101842

**Published:** 2023-01-23

**Authors:** eClinicalMedicine

January is Cervical Cancer Awareness Month, and this year marks just over 2 years since a landmark resolution to eliminate the cancer worldwide was announced by WHO. In November 2020, the Cervical Cancer Elimination Initiative (CCEI) was launched; an ambitious global strategy to eliminate cervical cancer as a public health problem, with the ultimate aim of reducing cervical cancer incidence to fewer than four women per 100,000 women-years worldwide. The strategy outlines clear targets for human papillomavirus (HPV) vaccination, cervical cancer screening, and effective treatment that must be met to show progress towards achieving this goal by 2030: 90% of girls are fully vaccinated with HPV vaccine by age 15 years; 70% of women are screened with a high-performance test by age 35 years and again by age 45 years; and 90% of women identified with cervical disease receive treatment. Despite 194 countries pledging to meet these targets, evidence suggests that large disparities in disease burden, access to HPV vaccination and screening between countries exist, with the COVID-19 pandemic exacerbating these disparities and disrupting access to preventive strategies. Are we on track to meet the targets of the CCEI, or do we need to accelerate our efforts further?

Worldwide, cervical cancer is the fourth most common cancer and the fourth most common cause of cancer death in women. Estimates suggest that the global age-standardised incidence of cervical cancer was 13.3 cases per 100,000 women-years in 2020, and the mortality rate was 7.2 deaths per 100 000 women-years. There are considerable disparities in incidence and mortality rates between countries and regions, and a clear inverse relationship between socioeconomic development and cervical cancer incidence and mortality rates. Unfortunately, the incidence of cervical cancer in most (93%) countries surveyed was found to exceed the threshold set by the WHO CCEI.

Almost all (99%) cervical cancers are caused by persistent infections with one of 12–14 carcinogenic HPV types, notably HPV 16 and 18, transmitted via sexual contact. HPV infection has also been implicated in the development of anogenital warts, squamous cell carcinomas of the anus, and cancers of the vulva, vagina, penis, oropharynx, and larynx. The discovery of HPV as a carcinogen has led to the development of safe and effective prophylactic HPV vaccines, with six vaccines having received marketing authorisation or WHO prequalification to date. The vital role of HPV vaccination as a primary cervical cancer prevention method was shown in an observational study showing an 87% reduction in cervical cancer incidence and a 97% reduction in grade 3 cervical intraepithelial neoplasia incidence in women aged 20–29 years who were offered the vaccine at age 12–13 years as part of the national HPV vaccination programme in England. Evidence also shows that quadrivalent and nonavalent HPV vaccines offer good protection against precancerous anogenital lesions and genital warts in men, indicating the benefits of extending national HPV vaccination programmes to boys as well as girls.

WHO recommends that the HPV vaccine be included in all national immunisation campaigns, and that prevention of cervical cancer is best achieved through vaccination of girls before they are sexually active. To date, 125 countries have introduced the HPV vaccine in their national immunisation programme for girls, with 47 countries extending the programme also to boys. However, this corresponds to only around a third of the global target population. Introduction of the vaccine in low-income and middle-income countries (LMICs), where 90% of deaths occur, remains slower than in high-income countries (HICs). Worryingly, declining trends in global HPV vaccine coverage have also been observed, with global coverage of the first dose of HPV vaccine among girls decreasing from 20% in 2019 to 15% in 2021. This coverage is far from the global target in girls of 90% outlined in the CCEI.

Cervical cancer is curable if detected early, usually through screening and ablative or excision treatment of pre-cancerous lesions, or referral to appropriate treatment facilities if invasive cervical cancer is suspected. WHO recommends using HPV DNA detection as the primary screening test, rather than visual inspection with acetic acid or cytology. Cervical samples can be self-collected or provider-collected and used in a screen-and-treat approach, or in a screen, triage, and treat approach. In a large review of cervical cancer screening programmes covering 202 countries and territories worldwide, around two-thirds of women aged 20–70 years were found to have never been screened for cervical cancer, despite most (69%) countries offering recommendations for screening. Highlighting the wide disparities in screening coverage worldwide, only 9–11% of women aged 30–49 years in LMICs found to have been screened during their lifetime compared with 83% of women in HICs. Extension of screening through self-sampling methods, which have comparable diagnostic accuracy to clinician-collected samples, could expand the reach of screening, particularly for underserved populations in LMICs or in areas of conflict. In England, where screening coverage has been declining over the past 20 years, just over 50% of eligible women reported preferring self-sampling over being tested by a clinician, indicating that this method could also help to improve screening coverage in HICs. Of note, WHO recognises the need for screening and treatment services to be more inclusive of intersex, gender fluid, and transgender people who have a cervix, and that more data are needed about the provision of services to these individuals.

The COVID-19 pandemic resulted in full or partial interruption to vaccination and cancer screening programmes worldwide, delaying access to vital preventive strategies. Additionally, considerable disparities between countries exist in terms of the availability and access to recommended HPV vaccination and cervical screening programmes. With an estimated 60 million preventable cervical cancer cases and 45 million preventable deaths over the next 100 years if the CCEI targets are met, it is imperative that we accelerate our efforts to ensure successful and equitable implementation of this important strategy worldwide.

